# Synthesis, Structure Activity Relationship Studies and Pharmacological Evaluation of 2-Phenyl-3-(Substituted Phenyl)-3H-Quinazolin-4-ones as Serotonin 5-HT_2_ Antagonists

**DOI:** 10.4103/0250-474X.58185

**Published:** 2009

**Authors:** N. Sati, S. Kumar, M. S. M Rawat

**Affiliations:** Department of Pharmaceutical Sciences, H. N. B. Garhwal University, Srinagar Garhwal-246 174, India; 1 College of Pharmacy, IFTM, Lodipur-Raipur, Delhi Road, Moradabad-244 001, India; 2 Department of Chemistry, H. N. B. Garhwal University, Srinagar Garhwal-246 174, India

**Keywords:** 5-HT_2_ antagonists, aryl quinazolines, quinazolin-4-ones, serotonin antagonists

## Abstract

Benzoyl chloride was added to the solution of anthranilic acid in pyridine to afford crude 2-phenyl-benzo[d][1, 3] oxazin-4-one (1). To the solution of compound 1 in dry toluene, various substituted anilines were added and the mixture refluxed for 8 h to afford 2-phenyl-3-(substituted phenyl)-3H-quinazolin-4-ones (2a-2f). All the compounds were obtained in solid state in yields varying between 40 to 70%. Spectral characterization included FTIR, ^1^H NMR and Electrospray MS. The synthesized compounds were screened for 5-HT_2_ antagonist activity. Some of the title compounds have been found to show significant 5-HT_2_ antagonist activity. The compound 2b, 3-(2-chlorophenyl)-2-phenyl-3H-quinazolin-4-one was the most potent derivative in the series of compound synthesized.

Serotonin receptors (5-HT_2_) receptors are of significant clinical interest because of their involvement in various disorders[1,2]. Centrally acting 5-HT_2_ antagonists have shown promising effects in animal models for anxiety[3], depression[4] and in certain drug abuse models[5–7]. In schizophrenic patients, improvement of negative symptoms[8] and extra pyramidal symptoms[9,10] has been demonstrated by 5-HT_2_ antagonists.

Quinazolines linked to aryl moiety are reported as anticonvulsant, antidepressant, tranquilizer and antagonist of 5-HT_2_ receptors[11–15]. In view of these observations, we herein report the synthesis of 2-phenyl-3-(substituted phenyl)-3H-quinazolin-4-ones and evaluate them for 5-HT_2_ antagonist activity. Our synthetic efforts were directed to find the effect of substitution in the phenyl ring on the antagonistic activity at 5-HT_2_ receptor.

Melting points of the synthesized compounds were determined by open capillary method and are uncorrected. Thin layer chromatography of synthesized compounds was performed on pre-coated silica gel G_254_ plates and visualized in iodine or UV. The IR spectra of synthesized compounds were recorded on Perkin-Elmer FTIR in potassium bromide discs. The proton nuclear magnetic resonance (^1^H NMR) was recorded in NMR Varian Mercury 300 MHz. The solvents used were DMSO-d_6_ and acetone. Chemical shifts are reported in δ ppm, downfield from tetramethylsilane (δ 0.00). Splitting patterns are designated as singlet (s), doublet (d) and multiplet (m). The electrospray mass spectra (ESMS) were recorded on a Micromass Quattro II triple quadrupole mass spectrometer. Elemental analysis was performed (C, H and N). All the target compounds and the reference olanzapine were orally administered (5-HTP was intraperitoneally administered). All protocols of animal experiments have been approved by Institutional Animal Ethics Committee. Antagonism of serotonin (5-HT_2_) receptor was studied by inhibition of L-5-hydroxytryptophan induced head twitches behaviour[16].

Group of six male Wistar rats (180-280 g) was used in this test procedure. Sixty minutes prior to scoring head twitch behaviour, L-5-hydroxy tryptophan (5-HTP) was administered intraperitoneally at dosage of 10 mg/kg. Thirty minutes following the injection, test compounds were administered orally. Control group were administered the appropriate vehicle. Sixty minutes post 5-HTP administration; each animal was individually placed in a clear plastic cage and observed for head twitch response[16]. The number of head twitches per animal was recorded over a 10 m interval and the total summed for each group. The percentage change from control for each group was then calculated. ED_50_ values were calculated by sigmoidal dose-response curve analysis using the program PRISM (Graph pad Software). P-value less than 0.05 (*P* < 0.05) was considered statistically significant.

The title compounds were synthesized as per Scheme 1, the procedural details are as follows; Phenyl-benzo[d][1,3]-oxazin-4-one (1) benzoyl chloride (0.21 mol) was added to the solution of anthranilic acid (0.01 mol) in pyridine drop-wise at room temperature with stirring for 30 min followed with the addition of 5% NaHCO_3_. The separated solid was filtered, washed with NaHCO_3_ and dried to afford crude 2-phenyl-benzo-[d][1,3]-oxazin-4-one (1), which was recrystallized in ethanol. Yield 63%, mp 120-121°, IR (KBr, cm^−1^) 3040 (-CH aromatic), 1764 (CO in lactone), 1315 (-CN in tert. amines), ^1^H NMR (δ ppm, DMSO-d_6_) 7.2-7.32 (m, 5H, phenyl at C_2_), 7.8-8.01 (m, 4H, C_5_, C_6_, C_7_ and C_8_ protons), ESMS: 224 (M+H)^+^, 146

**Scheme 1:**
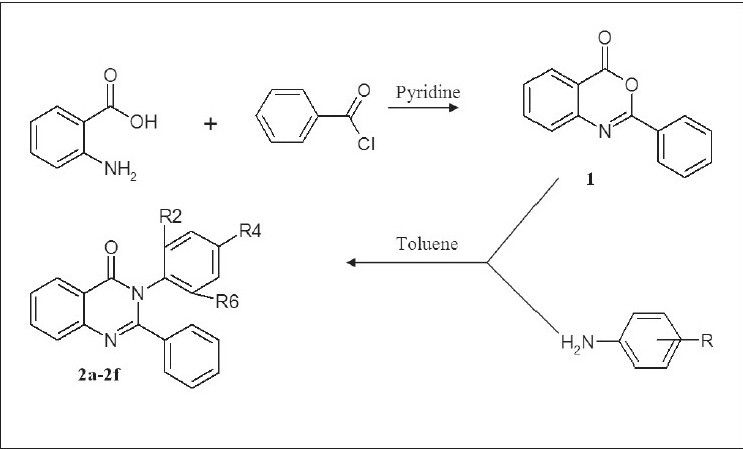
Synthesis of 2-phenyl-3-(substituted phenyl)-3h-quinazolin-4-ones

General method for the preparation of 2-phenyl-3-(substituted phenyl)-3H-quinazolin-4-ones (2a-2f) is as follows. To a solution of compound 1 in dry toluene, substituted anilines were added and the mixture refluxed for 8 h. The mixture was allowed to cool to room temperature and then poured in ice cold water. The precipitated solid was separated and dried to afford the target compounds 2-phenyl-3-(substituted phenyl)-3H-quinazolin-4-ones (2a-2f). All the compounds were recrystallized in methanol. The characterization and spectral data of the synthesized compounds is given in Tables 1 and 2, respectively.

**TABLE 1:** CHARACTERIZATION DATA OF THE TITLE COMPOUNDS

Compd.	R_2_	R_4_	R_6_	Mol. Formulae^a^	mp^b^	Yield^c^^d^	R_f_^e^
2a	H	H	H	C_20_H_14_N_2_O	116-118	53	0.56
2b	Cl	H	H	C_20_H_13_ClN_2_O	122-124	61	0.52
2c	H	F	H	C_20_H_13_FN_2_O	135-137	43	0.53
2d	CH_3_	H	CH_3_	C_22_H_18_N_2_O	159-161	51	0.63
2e	CH_3_	CH_3_	H	C_22_H_18_N_2_O	145-147	66	0.57
2f	OCH_3_	H	H	C_21_H_16_N_2_O_2_	142-144	69	0.60

a Elemental analyses results were within ± 0.4% of the calculated values

b Melting points are expressed in °C and are uncorrected

c Solvent for recrystallization is alcohol

d Yields are not optimized

e Mobile phase is 20 % EtOAc-benzene

**TABLE 2:** SPECTRAL CHARACTERIZATION DATA OF THE TITLE COMPOUNDS

Comp	IR^a^ (cm^−1^)	^1^H NMR^b^ (δ ppm)	ES MS (m/z)
2a	3030 (-CH aromatic), 1665 (CO in δ lactam), 1312 (-CN in tert. amines)	6.95-7.05 (m, 5H, phenyl at C_3_), 7.13-7.18 (m, 5H, phenyl at C_2_), 7.73-8.01 (m, 4H, C_5_, C_6_, C_7_ and C_8_ protons)	299, 221
2b	3029 (-CH aromatic), 1680 (CO in δ lactam), 1310 (-CN in tert. amines)	6.91-7.02 (m, 4H, phenyl at C_3_), 7.13-7.18 (m, 5H, phenyl at C_2_), 7.75-8.02 (m, 4H, C_5_, C_6_, C_7_ and C_8_ protons)	333, 255
2c	3021 (-CH aromatic), 1675 (CO in δ lactam), 1325 (-CN in tert. amines), 1050 (C-F)	6.93-7.05 (m, 4H, phenyl at C_3_), 7.15-7.19 (m, 5H, phenyl at C_2_), 7.8-8.04 (m, 4H, C_5_, C_6_, C_7_ and C_8_ protons)	317, 239
2d	3049 (-CH aromatic), 1668 (CO in δ lactam), 1305 (-CN in tert. amines)	2.34 (s, 3H, methyl at phenyl ring), 2.35 (s, 3H, methyl at phenyl ring), 7.04-7.08 (m, 3H, phenyl at C_3_), 7.11-7.15 (m, 5H, phenyl at C_2_), 7.74-8.1 (m, 4H, C_5_, C_6_, C_7_ and C_8_ protons)	327, 249
2e	3023 (-CH aromatic), 1673 (CO in δ lactam), 1319 (-CN in tert. amines)	2.33 (s, 3H, methyl at phenyl ring), 2.35 (s, 3H, methyl at phenyl ring), 7.03-7.07 (m, 3H, phenyl at C_3_), 7.12-7.19 (m, 5H, phenyl at C_2_), 7.7-8.04 (m, 4H, C_5_, C_6_, C_7_ and C_8_ protons)	327, 249
2f	3033 (-CH aromatic), 1667 (CO in δ lactam), 1310 (-CN in tert. amines)	3.83 (s, 3H, -OCH_3_ protons), 7.05-7.09 (m, 4H, phenyl at C_3_), 7.10-7.19 (m, 5H, phenyl at C_2_), 7.73-8.01 (m, 4H, C_5_, C_6_, C_7_ and C_8_ protons)	329, 251

a KBr pellet was used for determination

b Solvent used was dueterated acetone

Compound 1 showed IR absorption at 1764 cm^−1^ corresponding to carbonyl group stretching in lactones. ^1^H NMR spectral studies showed multiplets in the regions 7.2-7.3 δ and 7.8-8.01 δ, assigned respectively to protons of phenyl ring at C_2_ and the protons at C_5_, C_6_, C_7_ and C_8_. The ESMS of the compound 1 showed its (M+H)^+^ peak at 224.

The IR spectra of the title compounds 2a-2f showed characteristic absorption peaks in the range 1665-1680 cm^−1^, assigned to carbonyl stretching in δ lactams. The ^1^H NMR spectral studies showed multiplets in the range 6.91-7.09, 7.13-7.19 and 7.73-8.1δ, assigned respectively to phenyl at C_3_, phenyl at C_2_ and the protons at C_5_, C_6_, C_7_ and C_8_. The ES MS of title compounds showed their respective (M+H)^+^ peaks. All these observations confirmed the structures of the title compounds 2a-2f. Characterization data of the synthesized compounds is given in Table 1.

The propensity of title compounds to antagonize 5-HTP induced head twitch was evaluated and their respective ED_50_ values are reported in the Table 3. Our synthetic efforts in the series of compounds synthesized were directed to find the effect of electronic character of the substituent in phenyl ring on the affinity for 5-HT_2_ receptor. As is evident from Table 3, the efficacy of the compounds to antagonize the head twitch depended greatly on the nature of substituent. Of the synthesized compounds, not all were able to antagonize the head twitches induced by 5-HTP. The presence of electron withdrawing groups (Cl and F) at ortho- and para- positions lead to potent derivatives 2b and 2c. Substitution of the ortho- hydrogen by electron donating group (-OCH_3_) decreased the potency; however it was more potent than the unsubstituted compound 2a. Di-substitution with methyl at 2, 4 and 2, 6 positions yielded the least potent members of the series, i.e the compounds 2d and 2e. The most potent compound in the series was 3-(2-chlorophenyl)-2-phenyl-3H-quinazolin-4-one (compound 2b). Thus, the series synthesized afforded compounds having varying degree of affinity for the 5-HT_2_ receptor and offers interesting series of molecules to be worked upon for refinement of biological activity.

**TABLE 3:** INHIBITION OF HEAD TWITCHES AFTER ADMINISTRATION OF TEST AND REFERENCE COMPOUND

Compd.	ED_50_^a^ (mg / kg)	Compd.	ED_50_^a^ (mg / kg)
2a	28.63	2e	> 40
2b	15.35	2f	25.13
2c	17.19	Olanzapine	4.36
2d	> 40		

a ED_50_ was calculated by sigmoidal dose response curve analysis using the program PRISM (Graphpad Software) after oral administration of drug. *P*-value less than 0.05 (*P*<0.05) was considered statistically significant.
